# Short-term cultured, interleukin-15 differentiated dendritic cells have potent immunostimulatory properties

**DOI:** 10.1186/1479-5876-7-109

**Published:** 2009-12-18

**Authors:** Sébastien Anguille, Evelien LJM Smits, Nathalie Cools, Herman Goossens, Zwi N Berneman, Vigor FI Van Tendeloo

**Affiliations:** 1 University of Antwerp - Faculty of Medicine, Vaccine & Infectious Disease Institute (Vaxinfectio), Laboratory of Experimental Hematology, Universiteitsplein 1, B-2610 Wilrijk (Antwerp), Belgium; 2 Antwerp University Hospital, Center for Cell Therapy & Regenerative Medicine (CCRG), Wilrijkstraat 10, B-2650 Edegem (Antwerp), Belgium

## Abstract

**Background:**

Optimization of the current dendritic cell (DC) culture protocol in order to promote the therapeutic efficacy of DC-based immunotherapy is warranted. Alternative differentiation of monocyte-derived DCs using granulocyte macrophage colony-stimulating factor (GM-CSF) and interleukin (IL)-15 has been propagated as an attractive strategy in that regard. The applicability of these so-called IL-15 DCs has not yet been firmly established. We therefore developed a novel pre-clinical approach for the generation of IL-15 DCs with potent immunostimulatory properties.

**Methods:**

Human CD14^+ ^monocytes were differentiated with GM-CSF and IL-15 into immature DCs. Monocyte-derived DCs, conventionally differentiated in the presence of GM-CSF and IL-4, served as control. Subsequent maturation of IL-15 DCs was induced using two clinical grade maturation protocols: *(i) *a classic combination of pro-inflammatory cytokines (tumor necrosis factor-α, IL-1β, IL-6, prostaglandin E_2_) and *(ii) *a Toll-like receptor (TLR)7/8 agonist-based cocktail (R-848, interferon-γ, TNF-α and prostaglandin E_2_). In addition, both short-term (2-3 days) and long-term (6-7 days) DC culture protocols were compared. The different DC populations were characterized with respect to their phenotypic profile, migratory properties, cytokine production and T cell stimulation capacity.

**Results:**

The use of a TLR7/8 agonist-based cocktail resulted in a more optimal maturation of IL-15 DCs, as reflected by the higher phenotypic expression of CD83 and costimulatory molecules (CD70, CD80, CD86). The functional superiority of TLR7/8-activated IL-15 DCs over conventionally matured IL-15 DCs was evidenced by their *(i) *higher migratory potential, *(ii) *advantageous cytokine secretion profile (interferon-γ, IL-12p70) and *(iii) *superior capacity to stimulate autologous, antigen-specific T cell responses after passive peptide pulsing. Aside from a less pronounced production of bioactive IL-12p70, short-term *versus *long-term culture of TLR7/8-activated IL-15 DCs resulted in a migratory profile and T cell stimulation capacity that was in favour of short-term DC culture. In addition, we demonstrate that mRNA electroporation serves as an efficient antigen loading strategy of IL-15 DCs.

**Conclusions:**

Here we show that short-term cultured and TLR7/8-activated IL-15 DCs fulfill all pre-clinical prerequisites of immunostimulatory DCs. The results of the present study might pave the way for the implementation of IL-15 DCs in immunotherapy protocols.

## Background

Since their discovery by Steinman and Cohn in 1973, dendritic cells (DCs) have been recognized as the strategic orchestrators of the innate and adaptive immune system [1-3]. Although our knowledge of DC biology is still expanding, several concepts are yet well established [3,4]. Immature DCs are known to be the vigilant sentinels of the human immune system; they relentlessly screen the environment for the presence of antigen and are highly capable of antigen uptake [4,5]. Mature DCs are able to present the processed antigens via major histocompatibility complexes (MHC) to T cells after their migration to secondary lymphoid organs. This process of DC-mediated migration is regulated by multiple factors, but expression of the chemokine receptor CCR7 is recognized to play a pivotal role [6]. In the lymph nodes, three signals are required for the formation of an optimal immunological synapse between DCs and T cells and for the induction of desired T helper type 1 (T_h_1) immune response: *(1) *recognition of MHC-presented antigens by T cell receptors, *(2) *delivery of costimulatory signals via the CD80/CD86-CD28 pathway, and *(3) *secretion of interleukin (IL)-12p70 by DCs after CD40/CD40 ligand signalling [5].

Since DCs are key regulators of the human immune system, their use under the form of a cellular vaccine is an attractive strategy for the treatment of cancer and infectious diseases [3]. Since the results of the first clinical DC vaccine trial were published in 1996 [7], the field of DC-based immunotherapy has been increasingly translated into clinical practice, as evidenced by the growing number of clinical studies. To date, more than 100 trials have been performed or are currently ongoing to evaluate the effect of DC vaccines in a wide variety of disease states, with a main focus on the treatment of cancer [4].

While CD34^+ ^bone marrow progenitor cells and circulating blood myeloid DCs have been applied as DC precursors in some clinical studies, the vast majority of DCs used for vaccination purposes are derived from autologous peripheral blood monocytes [8]. The classic strategy for the *ex vivo *generation of monocyte-derived DCs consists of a two-step culture protocol, in which monocytes are differentiated towards immature DCs, followed by the induction of DC maturation. The total *in vitro *culture duration lasts one week, 5-6 days for DC differentiation and 1-2 days for subsequent DC maturation [5,9,10]. However, there is an increasing body of evidence that mature monocyte-derived DCs can be generated even after short-term cell culture for 2-3 days [9,11-15]. As compared to the traditional 7-day approach, rapid expansion of DCs is associated with several advantages; it simplifies the laborious and time-consuming process of DC manufacturing and it reduces the actual risk of microbial contamination related to *in vitro *culture [10,15]. Moreover, short-term cultured DCs exhibit equal or superior functional DC characteristics compared to their conventional long-term counterparts [13,14]. Previous work has already demonstrated the feasibility of short-term culture of monocyte-derived DCs differentiated in the presence of granulocyte macrophage colony-stimulating factor (GM-CSF) and IL-4 (IL-4 DCs) [12-16].

Alternative differentiation of monocyte-derived DCs using a combination of GM-CSF and IL-15 has recently gained increasing interest. Interleukin-15 is a pleiotropic cytokine that plays a pivotal role in the generation of antigen-specific CD8^+ ^T lymphocytes [17-19], the induction of memory CD8^+ ^T cell immunity [20] and natural killer (NK) cell activation [21]. Interleukin-15 differentiated DCs (IL-15 DCs) have been previously described to exhibit a distinct Langerhans cell(LC)-like phenotype and to possess unique immunostimulatory properties [22,23]. This was more recently supported by the demonstration that IL-15 DCs are endowed with a superior capacity to induce antigen-specific cellular immune responses in an *in vitro *melanoma tumor model [24,25]. These promising results make IL-15 DCs a qualified candidate for application in DC-based tumor immunotherapy [4,26].

In addition, the Toll-like receptor (TLR) signal transduction pathway has recently emerged as an attractive alternative for the induction of DC maturation [10,27,28]. Toll-like receptors recognize pathogen-derived signals, such as microbial constituents (viral or bacterial-derived proteins, RNA or DNA) [29,30]. Monocyte-derived DCs are known to express a series of TLRs, either on their cell surface (TLR2, TLR4) or intracellularly (TLR3, TLR7, TLR8 and TLR 9) [27]. Recent studies have suggested that DC maturation using TLR3 or TLR7/8 ligands in association with prostaglandin E_2 _(PGE_2_) results in the generation of DCs that, besides migratory properties, possess the desired capacity to produce T_h_1-polarizing cytokines such as IL-12p70 [31-33]. In the context of cancer immunotherapy, T_h_1 polarization is considered *conditio sine qua non *for the induction of anti-tumor cytotoxic immune responses. However, despite the discovery of TLR ligands as powerful DC maturation agents, a non-TLR ligand-based maturation cocktail is currently regarded as the 'gold standard' for the induction of DC maturation in clinical trials. This widely adopted maturation cocktail was first described by Jonuleit *et al. *and is composed of the pro-inflammatory cytokines tumor necrosis factor (TNF)-α, IL-1β, IL-6 and PGE_2 _[34]. Prostaglandin E_2 _is generally believed to be indispensable for potentiating the migratory potential of DCs [35,36], but hampered IL-12p70 production is considered to be its main drawback [37-39].

In view of the consideration that short-term DC culture, differentiation with IL-15 and TLR-induced maturation are proposed as separate attractive strategies to optimize the immunogenicity of clinical DC vaccination, we sought to determine whether an integration of these approaches is feasible and results in the generation of potent immunostimulatory DCs. We therefore examined the effect of culture duration on IL-15 DC phenotype and function by comparing short-term and long-term culture protocols. In addition, we evaluated the effect of two different maturation procedures on IL-15 DCs, juxtaposing the traditional pro-inflammatory cytokine combination with a clinical grade available maturation cocktail that includes a TLR7/8 ligand (resiquimod; R-848).

## Methods

### Generation of immature DCs

Peripheral blood mononuclear cells (PBMCs) were isolated from healthy donor buffy coat preparations using a standard density-gradient centrifugation technique (Ficoll-Paque™ PLUS, GE Healthcare; Diegem, Belgium). The freshly isolated PBMC-fraction was instantly used for immunomagnetic cell selection of monocytes with CD14 microbeads (Miltenyi Biotec; Amsterdam, The Netherlands). The CD14-depleted cell fraction, composed of peripheral blood lymphocytes (PBLs), was immediately cryopreserved in freezing solution containing 90% fetal calf serum (Perbio Science; Erembodegem, Belgium)/10% dimethyl sulfoxide (Sigma-Aldrich; Bornem, Belgium) and stored at -80°C until use. The positively selected cell population (mean purity of CD14^+ ^monocytes ± SD: 96.7 ± 1.5%), was subsequently used for the *in vitro *generation of DCs. For this purpose, monocytes were resuspended in RPMI 1640 culture medium (BioWhittaker; Verviers, Belgium) supplemented with 2.5% heat-inactivated human AB serum and seeded in 6-well culture plates (Corning Life Sciences; Schiphol-Rijk, The Netherlands) at a final concentration of 1-1.2 × 10^6^/mL. Monocytes were cultured with 800 IU/mL GM-CSF (Gentaur; Brussels, Belgium) and 20 ng/mL IL-4 (Gentaur; Brussels, Belgium) or 200 ng/mL IL-15 (Immunotools; Friesoythe, Germany) in order to generate immature IL-4 DCs and IL-15 DCs, respectively (Table 1).

**Table 1:** Differentiation and maturation procedures used in the present study.

**Dendritic cell differentiation**		
		
***IL-4 differentiated dendritic cells ***(IL-4 DCs)		
GM-CSF	800 IU/mL	Gentaur, Brussels, Belgium
IL-4	20 ng/mL	R&D Systems, Minneapolis, USA
***IL-15 differentiated dendritic cells ***(IL-15 DCs)		
GM-CSF	800 IU/mL	Gentaur, Brussels, Belgium
IL-15	200 ng/mL	Immunotools, Friesoythe, Germany
		
**Dendritic cell maturation**		
		
***cc-mDC ***(conventional maturation cocktail, according to Jonuleit *et al. *[34])		
TNF-α	10 ng/mL	Biosource, Nivelles, Belgium
IL-1β	10 ng/mL	R&D Systems, Minneapolis, USA
IL-6	15 ng/mL	Biosource, Nivelles, Belgium
PGE_2_	1 μg/mL	Pfizer, Puurs, Belgium
		
***TLR-mDC ***(TLR7/8 agonist-based maturation cocktail)		
R-848 (Resiquimod)	3 μg/mL	Alexis Biochemicals, San Diego, USA
TNF-α	2.5 ng/mL	Biosource, Nivelles, Belgium
IFN-γ	5000 IU/mL	Immunotools, Friesoythe, Germany
PGE_2_	1 μg/mL	Pfizer, Puurs, Belgium

### Induction of DC maturation

Two different maturation cocktails were used for the induction of DC maturation. The conventionally applied combination of pro-inflammatory cytokines, first described by Jonuleit *et al. *[34], was compared with a TLR7/8 agonist-based maturation cocktail. Table 1 provides an overview of the composition of the different maturation cocktails used in this study (Table 1). The resultant mature DCs were harvested 24 hr after addition of the maturation agents.

### Duration of *in vitro *culture

Short-term *versus *long-term culture protocols were performed in order to determine the effect of culture duration on IL-15 DC phenotype and function. Short-term DCs were cultured for two days and subsequently matured for another 24 hr. Likewise, the long-term DC culture protocol included a six-day period for the generation of immature DCs followed by one day to obtain complete maturation.

### Flow cytometric immunophenotyping

Immunofluorescent staining of cell surface antigens was performed using a panel of fluorescein isothiocyanate (FITC)- or phycoerythrin (PE)-conjugated monoclonal antibodies (mAb): CD1a (FITC, clone HI149), CD14 (FITC, clone MϕP9), CD40 (PE, clone 5C3), CD56 (FITC, clone NCAM16.2), CD70 (PE, clone Ki-24), CD80 (PE, clone L307.4), CD83 (PE, clone HB15e), CD86 (FITC, 2331 [FUN-1]), CD207/Langerin (PE, clone DCGM4), CD209/DC-SIGN (FITC, clone DCN46) and CCR7 (PE, clone 150503). All monoclonal antibodies were purchased from BD Biosciences (Erembodegem, Belgium), except for CD83 mAb (Invitrogen; Camarillo, CA, USA), CD207 mAb (Beckman Coulter; Marseille, France), and CCR7 mAb (R&D Systems; Minneapolis, MN, USA). Corresponding species- and isotype-matched antibodies were used as controls. Propidium iodide (PI; Sigma-Aldrich) was included in the analysis to discriminate between viable and dead cells. Data acquisition was performed on a FACScan™ multiparametric flow cytometer (BD Biosciences).

### FITC-dextran endocytosis assay

The mannose receptor-mediated endocytosis of FITC-labeled dextran particles (MW 40 kDa; Sigma-Aldrich) was determined by co-incubation of 0.4 × 10^6 ^immature DCs with 100 μg/mL FITC-dextran at 37°C. Parallel experiments were carried out at 4°C to determine the non-specific FITC-dextran uptake (negative controls). After 60 minutes, internalization of FITC-dextran was stopped by washing the cells twice with ice-cold phosphate-buffered saline (PBS; Gibco Invitrogen; Paisley, UK). The endocytic capacity was subsequently analyzed by flow cytometric quantitation of the specific FITC fluorescence signal intensity.

### Transwell™ chemotaxis assay

The migratory potential of IL-15 DCs was determined by a chemotaxis assay using 24-well culture plates carrying polycarbonate membrane-coated Transwell™ permeable inserts (5 μm pore size; Costar). First, the lower plate chambers were filled with 600 μL DC culture medium per well. The CCR7 ligand 6Ckine/CCL21 (R&D Systems) served as chemotactic agent and was added to the lower well at an optimal concentration of 100 ng/mL. Next, DCs (1.0 × 10^5 ^cells) were seeded on each Transwell™ insert in a total volume of 100 μL DC culture medium and allowed to migrate to the lower compartments for 180 min in a humidified 37°C/5% CO_2 _incubator (*chemokine-driven migration*). Parallel control experiments were conducted in the absence of CCL21 to assess the spontaneous cell migration (*negative control*) or by transferring all cells (1.0 × 10^5^) to the lower well in order to determine the maximum possible DC yield (*positive control*). Thirty minutes prior to harvest, 5 mM EDTA (Merck; Darmstadt, Germany) was added to the lower compartments to detach the adherent, transmigrated cells. Finally, the cells from each lower well were collected, centrifuged and concentrated to a final sample volume of 200 μL. Cells were counted in duplicate by flow cytometric analysis at a fixed flow rate during a defined time period of 60 sec (counts per minute; cpm). DC migration was expressed using the following equation:

### Cytokine secretion profile

The cytokine secretion profile of the different DC subsets was assessed by a multiplex immunoassay (MIA). Briefly, mature DCs were harvested, extensively washed and resuspended in fresh DC culture medium (5.0 × 10^5 ^cells/mL), not containing any exogenous growth factor or cytokine. After 24 hr of incubation, culture supernatants were analyzed for the presence of 11 different pro-inflammatory and T_h_1/T_h_2-polarizing cytokines using a commercially available MIA kit (FlowCytomix human T_h_1/T_h_2 11plex kit, Bender Medsystems; Vienna, Austria), according to the manufacturer's instructions.

### IL-12p70 ELISA following CD40 ligation ("signal-3 assay")

Human CD40 ligand (CD40L)-expressing mouse 3T3 fibroblasts (kindly provided by Dr K. Thielemans, Free University Brussels, Brussels, Belgium) were suspended in a 48-well culture plate at a concentration of 2.5 × 10^5 ^cells per well and incubated overnight at 37°C to allow stable reattachment on the bottom surface of the well. The next day, mature DCs were seeded on the 3T3 feeder cell layer at a density of 5.0 × 10^5 ^cells per well in a total volume of 1 mL fresh DC culture medium. After 24 hr of co-incubation at 37°C, supernatants were carefully collected and stored frozen at -20°C until further use. The production of bioactive IL-12p70 was next determined using a commercially available standard sandwich ELISA kit (eBioscience; San Diego, CA, USA).

### Autologous T cell stimulation capacity

To assess their autologous T cell stimulation capacity, mature DCs were pulsed with a panel of 32 MHC class I-restricted antigen epitopes derived from cytomegalovirus, Epstein-Barr virus and influenza virus, designated to as CEF peptide pool. The CEF peptide pool was obtained through the NIH AIDS Research & Reference Reagent Program (Division of AIDS, NIAID, NIH; Germantown, MD, USA) and used at a total concentration of 1 μg/μL.

Peptide-pulsed DCs were subsequently co-incubated with autologous PBLs at a 1:10 ratio in RPMI supplemented with 1% human AB serum. By day 7 of coculture, PBLs were harvested and restimulated with the CEF peptide pool for an additional 6 hr. A peptide mixture composed of human papilloma virus (HPV) type 16 E7 peptides served as negative control. The HPV peptide pool consisted of nine HPV_16 _E7 18- to 20-mer peptides (each overlapping by 10 amino acids), spanning the full length of the HPV_16 _E7 protein (1 μg/μL; AC Scientific; Duluth, GA, USA). Antigen-specific interferon (IFN)-γ secretion following peptide stimulation was determined by ELISA (Peprotech; Rocky Hill, NJ, USA) as per the manufacturer's protocol.

For intracellular staining (ICS) of IFN-γ, PBLs (1 × 10^6^) were harvested after coculture with autologous CEF-pulsed DCs and subjected to a similar antigen stimulation protocol. Brefeldin A (1 μL; GolgiPlug™, BD Biosciences) was added during the stimulation period in order to sequester IFN-γ intracellularly. After 6 hr, PBLs were washed with PBS containing 1% bovine serum albumin and 0.1% sodium azide. Prior to the fixation and permeabilization procedure, cell surface staining for CD8 (PE, clone SK1, BD Biosciences) and CD3 (PerCP, clone SK7; BD Biosciences) was performed as described above. Next, cells were fixated and permeabilized using BD FACS™ lysing solution (1×) and permeabilizing solution 2 (1×). Intracellular staining was performed using IFN-γ mAb (15 ng per 1 × 10^6 ^cells; FITC, clone B27, BD Biosciences). Cells were subsequently incubated overnight at 4°C prior to flow cytometric analysis.

### mRNA electroporation of IL-15 DCs

DNA transcription templates encoding the enhanced green fluorescent protein (eGFP) and influenza virus M1 matrix protein were respectively derived from the pGEM4Z/EGFP/A64 (kindly provided by Dr. E. Gilboa, then at Duke University Medical Center, Durham, NC, USA) and pGEM4Z/M1/A64 (kindly provided by Dr. A. Steinkasserer, University of Erlangen, Erlangen, Germany) plasmid vectors, according to our previously described protocol [9]. Subsequent *in vitro *transcription of mRNA was performed using a commercially available T7 polymerase-based transcription kit (Ambion; Austin, TX, USA), following the manufacturer's instructions.

Mature IL-15 DCs were harvested and washed twice in serum-free IMDM culture medium (Cambrex Bio Science; Verviers, Belgium) and Opti-MEM I medium (Gibco Invitrogen), respectively. Next, 1 × 10^6 ^DCs were resuspended in a total volume of 200 μL Opti-MEM I and transferred to a 4.0-mm electroporation cuvette (Cell Projects; Harrietsham, UK). After addition of *in vitro *transcribed eGFP mRNA (20 μg) or M1 mRNA (10 μg), electroporation was performed using a Gene Pulser Xcell™ device (Bio-Rad Laboratories; Hercules, CA, USA) at predefined settings (300 V; 150 μF; 7.0 ms). The mRNA electroporation efficiency was assessed by flow cytometric analysis of the eGFP expression levels at different time points post-electroporation (4 hr, 24 hr, 48 hr). Propidium iodide was included in the assay to determine the post-electroporation cell viability.

### Antigen-presenting function of mRNA-electroporated IL-15 DCs

HLA-A*0201^+ ^IL-15 DCs were electroporated with M1-encoding mRNA and cocultured at a 1:10 ratio with autologous PBLs in 24-well polystyrene culture plates. Six days after initiation of the coculture experiments, PBLs were harvested and counted using an automatic hemocytometer.

To determine the presence of M1-specific CD8^+ ^T lymphocytes, 1 × 10^6 ^PBLs were stained with anti-CD8 (FITC, clone SK1; BD Biosciences) and PE-conjugated HLA-A*0201 tetramer loaded with the influenza virus M1 matrix peptide (GILGFVFTL; kindly provided by Prof. P. Van der Bruggen, Ludwig Institute for Cancer Research, Brussels, Belgium). A dump channel (PerCP) was included to enhance the specificity of the tetramer assay.

Concomitantly, a fraction of the cocultured PBLs was subjected to antigen restimulation using two HLA-A*0201 restricted, virus-specific epitopes: the influenza matrix protein M1 peptide (M1_58-66 _[GILGFVFTL]; Eurogentec; Seraing, Belgium) and an irrelevant peptide fragment derived from carcinoembryonic antigen (CEA_571-579 _[YLSGANLNL]; Eurogentec). Both peptides were used at a final concentration of 1 μg/mL. The duration of antigen stimulation was 4 hours, after which the level of IFN-γ producing CD8^+ ^T cells was determined using a similar ICS protocol as described above.

### Data mining and statistical analysis

Flow cytometric data analysis was performed using FlowJo version 8.4.4 (TreeStar; San Carlos, CA). Phenotypic results were expressed as Δ mean fluorescence intensity (MFI), *i.e. *the difference between the MFI values obtained from the specific mAb and the corresponding isotype-matched control, or calculated as a percentage of positive cells using the SuperEnhanced D-max or Overton histogram subtraction methods. GraphPad Prism 4.0 software (GraphPad Software; San Diego, CA, USA) was used for graphical data representations and statistical computations. Statistical analysis was performed using Student's *t*-test or repeated-measures ANOVA with Bonferroni's post-hoc testing, where appropriate. Any *P*-value < 0.05 was considered statistically significant.

## Results

### Immature IL-15 DCs display a unique phenotype

**Figure 1:**
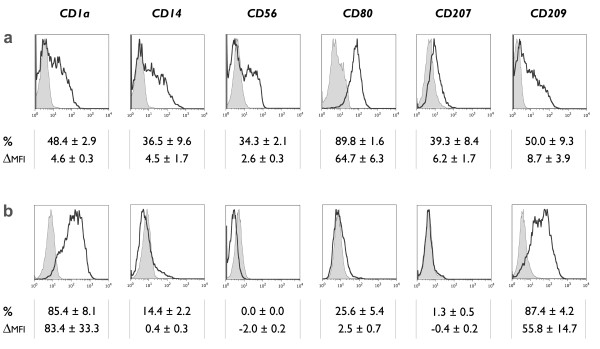
**Phenotypic characteristics of immature IL-15 DCs**. Immature DCs were analyzed by flow cytometry for the expression of CD1a, CD14, CD56, CD80, CD207 (Langerin) and CD209 (DC-SIGN). The histograms represent the expression of the indicated cell surface antigens (bold-line histograms) and the corresponding isotype controls (grey-filled histograms). The mean ± SEM percentage of positive cells (%) and delta MFI ± SEM (ΔMFI) were calculated as specified in the "Methods" section (*n *= 3-6). **(a)** Phenotype of monocyte-derived DCs generated in the presence of GM-CSF + IL-15 and harvested at the immature stage 2-3 days after initiation of the DC culture. **(b)** Corresponding phenotypic profile of conventional immature DCs, differentiated in the presence of GM-CSF + IL-4.

Immature monocyte-derived DCs differentiated for 2 days in the presence of GM-CSF and IL-15 were evaluated for the phenotypic expression of CD1a, CD14, CD56, CD80, CD207 (Langerin) and CD209 (DC-SIGN) (Figure 1). The monocyte marker CD14 was found to be rapidly downregulated on immature IL-15 DCs, although a persistent basal expression level could still be observed (Figure 1a). This finding contrasts with the near-absence of CD14 on conventional immature IL-4 DCs (Figure 1b). The incomplete disappearance of CD14 on the cell surface of IL-15 DCs could not be explained by the short-term duration of culture (2 days), since long-term cultured IL-15 DCs (6 days) displayed even higher levels of CD14 (data not shown). As opposed to CD14, the cell surface expression of DC-related molecules CD1a and CD209 (DC-SIGN) was found to be more pronounced on IL-4 DCs. Conversely, IL-15 DCs expressed the costimulatory molecule CD80 at the immature stage whereas IL-4 DCs did not. In addition, IL-15 DCs showed a unique phenotype with partial positivity for CD207, a LC-related surface antigen, and CD56, a marker with a dominant expression on NK cells.

### TLR7/8-activated IL-15 DCs acquire a mature phenotype

**Figure 2:**
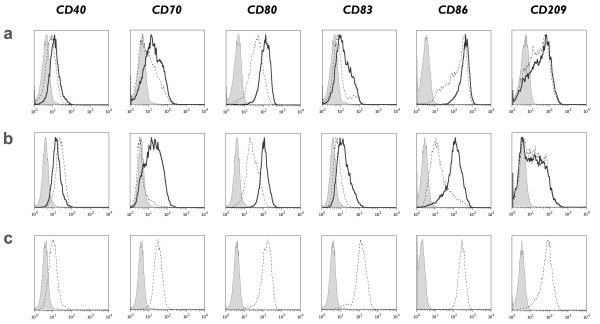
**Phenotypic characteristics of mature IL-15 DCs**. Immunophenotypic expression of CD40, CD70, CD80, CD83, CD86 and CD209 (DC-SIGN) by **(a) **short-term cultured IL-15 DCs, **(b) **long-term cultured IL-15 DCs and **(c) **conventional IL-4 DCs after maturation induction with either a classic maturation cocktail (cc-mDC; dashed-line histograms) or a TLR7/8 ligand-containing mixture (TLR-mDC; bold-line histograms). Isotype controls are represented by the grey-filled histograms. A detailed overview of the flow cytometry data of mature DCs is provided in "Additional file 1" (*n *= 4).

We first assessed the phenotypic differences between mature IL-15 DCs (Figure 2a and 2b) and "standard" mature IL-4 DCs (Figure 2c). As shown in figure 2, maturation of IL-15 DCs and IL-4 DCs was associated with an upregulation of CD40, CD80, CD86 and of the DC maturation marker CD83. The most striking difference between IL-15 DCs and conventionally matured IL-4 DCs was the higher level of CD83 expression in the latter DC subset, consistent with a more mature phenotype (Figure 2; Additional File 1).

Divergent phenotypic results were obtained with the 2 protocols used for the induction of IL-15 DC maturation: *(i) *a classic combination of pro-inflammatory cytokines (cc-mDC) versus *(ii) *a TLR7/8 ligand-containing maturation cocktail (TLR-mDC). As shown in Figure 2, the costimulatory molecules CD70, CD80 and CD86 were expressed at consistently higher levels upon activation of IL-15 DCs with the TLR7/8 agonist-based cocktail (TLR-mDC) as opposed to the conventional cytokine cocktail (cc-mDC). Moreover, a more profound maturation state was reached in TLR7/8-matured IL-15 DCs. This was reflected by the increased surface expression of CD83. The low expression of costimulatory molecules and CD83 after maturation of IL-15 DCs with the cytokine cocktail (cc-mDC) sharply contrasted with the effects of this maturation cocktail on IL-4 DCs (Figure 2; Additional file 1).

We next examined the effect of culture duration on the phenotype of mature IL-15 DCs. No apparent differences were observed between short-term (Figure 2a) and long-term cultured IL-15 DCs (Figure 2b), with the exception of CD86 which was found to be more pronounced in short-term cultured IL-15 DCs. The cell surface expression level of CD83 was independent of the duration of DC culture, suggesting that an equal maturation level can be obtained after short-term culture of IL-15 DCs.

A detailed overview of the phenotypic characteristics of mature IL-15 DCs is provided in "Additional File 1".

### Immature IL-15 DCs are capable of phagocytosis

**Figure 3:**
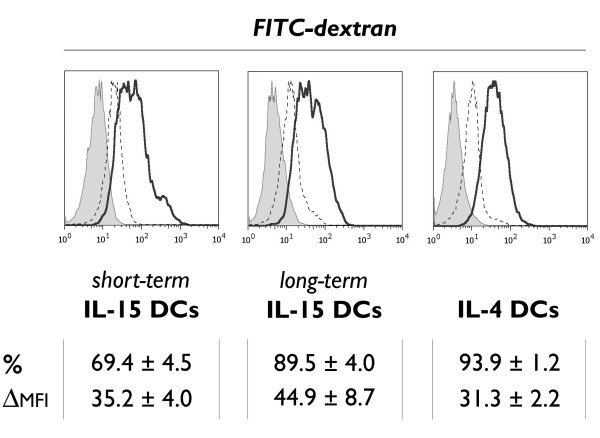
**Mannose receptor-mediated endocytosis of FITC-dextran particles**. Histogram overlays depicting the *in vitro *uptake of FITC-dextran molecules by immature DCs, respectively short-term cultured IL-15 DCs (left), long-term cultured IL-15 DCs (middle) and control IL-4 DCs (right). The FITC-dextran endocytosis at 37°C (bold-line histograms) is compared to the non-specific fluorescence at 4°C (dashed-line histograms) and to the autofluorescence from unlabeled samples (grey-filled histograms), as described in "Methods". The uptake of FITC-dextran was quantified as mean ± SEM percentage of FITC-dextran positive cells (%) and as delta MFI ± SEM (ΔMFI), which was calculated by subtracting the MFI value of the non-specific FITC-dextran uptake at 4°C from the MFI value obtained at 37°C (*n *= 3).

Immature IL-15 DCs were examined for their intrinsic phagocytosis capacity using a FITC-dextran endocytosis assay. Both short-term and long-term cultured IL-15 DCs showed a high potential for FITC-dextran phagocytosis, as reflected by the average number of dextran^+ ^cells and the mean fluorescence intensity of the FITC signal (Figure 3). The 1-hr FITC-dextran uptake did not differ significantly between both IL-15 DC subsets, and was found to be comparable to that of immature IL-4 DCs. Mature DCs displayed a reduced phagocytosis capacity compared to their immature counterparts (data not shown).

### Migratory potential of IL-15 DCs

The migratory properties of immature IL-15 DCs (iDC), conventionally matured IL-15 DCs (cc-mDC) and TLR7/8-matured IL-15 DCs (TLR-mDC) were compared by assessment of their CCR7 expression pattern and their *in vitro *migratory potential using a standard Transwell™ chemotaxis assay.

**Figure 4:**
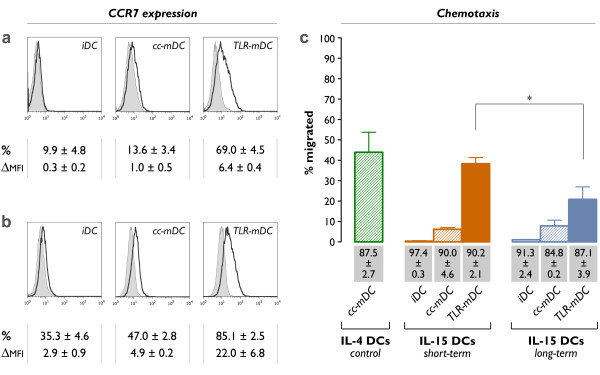
**CCR7 expression and migratory capacity**. Histogram overlays comparing the CCR7 expression on **(a) **short-term cultured and **(b) **long-term cultured IL-15 DCs, either at the immature stage (*iDC*) or at the mature stage (*cc-mDC*: + TNF-α, IL1β, IL-6 and PGE_2 _for the last 24 hours; *TLR-mDC*: + R-848, IFN-γ, TNF-α and PGE_2 _for the last 24 hours). Bold-line histograms represent the CCR7-specific staining, whereas the corresponding isotype controls are indicated by grey-filled histograms (*n *= 3). **(c) **Migration of the indicated DC subsets towards CCL21 in a Transwell chemotaxis assay. The mean ± SEM percentages of migrated cells after 180 min were calculated according to the formula specified in "Methods" (*n *= 3-6; *, *P *= 0.01). The values shown in the grey bars represent the cell viabilities of the different DC subsets (mean ± SEM; *n *= 3-6).

The phenotypic analysis revealed near-absent expression of CCR7 in immature IL-15 DCs, both after short-term (Figure 4a) and long-term DC culture (Figure 4b). Consequently, immature IL-15 DCs were unable to migrate to the secondary lymph node chemokine CCL21 in a Transwell™ chemotaxis assay (Figure 4c). Dendritic cells matured with the conventional mixture of pro-inflammatory cytokines (cc-mDC) displayed only weak CCR7 positivity. In line with their low CCR7 surface expression, we observed no relevant CCR7-driven migration by short-term and long-term conventionally matured IL-15 DCs (Figure 4c). Conversely, a marked up-regulation of the CCR7 cell surface expression was noted upon maturation of IL-15 DCs with the TLR7/8 agonist-based cocktail (TLR-mDC). Accordingly, TLR7/8-matured IL-15 DCs were endowed with potent migratory activity in the chemotaxis assay. The increased CCR7 expression state in long-term cultured TLR7/8-matured IL-15 DCs was not associated with a better migratory response as compared to the short-term cultured counterparts. As shown in figure 4c, a superior chemotactic potential was observed in short-term cultured TLR7/8-matured IL-15 DCs; their *in vitro *migratory behaviour was virtually comparable to that of standard IL-4 DCs (Figure 4c; IL-4 cc-mDC *vs. *short-term IL-15 TLR-mDC: *P *= 0.62).

### Cytokine secretion profile of IL-15 DCs

The MIA technique was used to assess the 24-hr cytokine secretion profile of mature IL-15 DCs and IL-4 DCs. As indicated in Table 2, the expression of a panel of 11 T_h_1/T_h_2-polarizing and pro-inflammatory cytokines was analyzed. Maturation of IL-15 DCs was induced using two different protocols, as described above (cc-mDC and TLR-mDC).

**Table 2:** Cytokine secretion profile of mature DCs.

	short-term IL-15 DCs		long-term IL-15 DCs		IL-4 DCs
			
	*cc-mDC*	*TLR-mDC*	*cc-mDC*	*TLR-mDC*	*cc-mDC*
	*(pg/mL)*	*(pg/mL)*	*(pg/mL)*	*(pg/mL)*	*(pg/mL)*
**Typical T_h_1-polarizing**					
*IL-12p70*	0 ± 0	0 ± 0	0 ± 0	0 ± 0	0 ± 0
*IFN-γ*	0 ± 0	3671 ± 394	0 ± 0	4510 ± 686	0 ± 0
*IL-2*	0 ± 0	0 ± 0	0 ± 0	0 ± 0	0 ± 0
					
**Typical T_h_2-polarizing**					
*IL-4*	0 ± 0	0 ± 0	0 ± 0	0 ± 0	0 ± 0
*IL-5*	9 ± 5	6 ± 6	0 ± 0	0 ± 0	11 ± 6
*IL-10*	0 ± 0	4 ± 4	0 ± 0	50 ± 50	0 ± 0
					
**Pro-inflammatory**					
*TNF-α*	281 ± 124	1132 ± 551	1078 ± 268	4424 ± 1446	39 ± 16
*TNF-β*	0 ± 0	0 ± 0	0 ± 0	0 ± 0	0 ± 0
*IL-1β*	60 ± 55	0 ± 0	146 ± 64	55 ± 33	71 ± 45
*IL-6*	1065 ± 102	8163 ± 3246	1660 ± 78	13391 ± 2732	793 ± 51
*IL-8*	9914 ± 986	6109 ± 2468	3876 ± 483	2061 ± 109	9495 ± 2289

*Abbreviations used*: short-term: 3-day culture; long-term: 7-day culture; cc-mDC: conventional maturation cocktail (TNF-α, IL1β, IL-6 and PGE_2_); TLR-mDC: TLR7/8 agonist-based maturation cocktail (R-848, IFN-γ, TNF-α and PGE_2_). Results are expressed as mean ± SEM (pg/mL).

**Figure 5:**
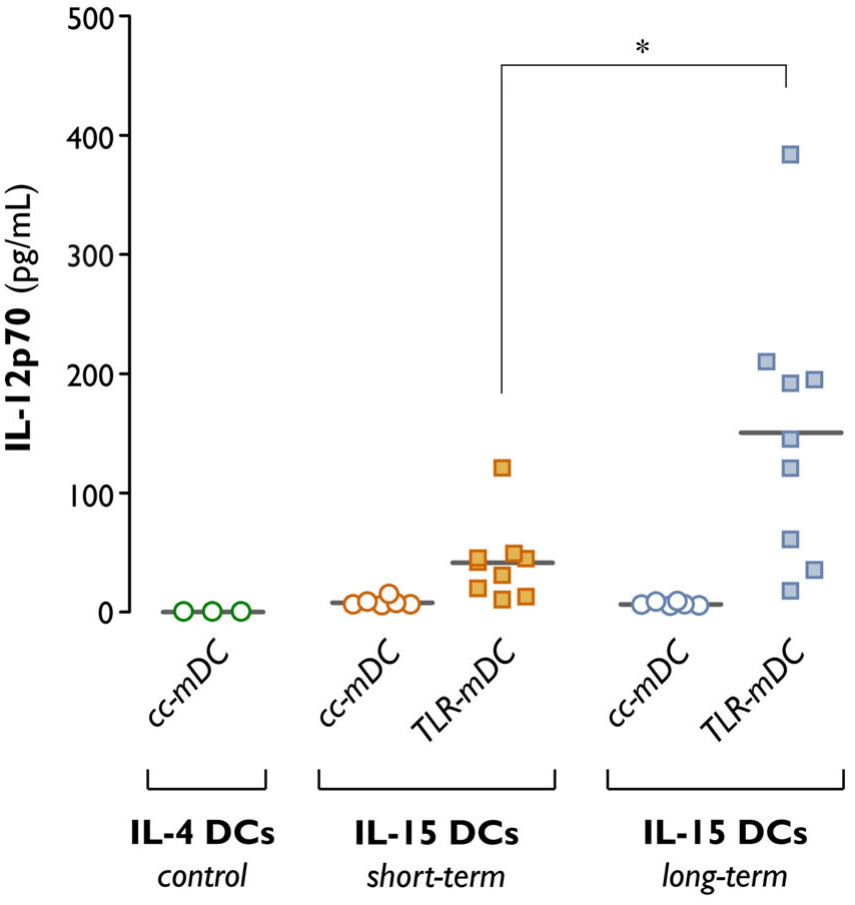
**IL-12p70 production following CD40 ligation ("signal-3 assay")**. Dendritic cells were differentiated in the presence of GM-CSF + IL-4 for 6 days (control IL-4 DCs), or in the presence of GM-CSF + IL-15 for 2 days (short-term IL-15 DCs) or 6 days (long-term IL-15 DCs). Dendritic cell maturation was induced by addition of two different maturation cocktails 24 hr prior to DC harvest (*cc-mDC*: TNF-α, IL1β, IL-6 and PGE_2_; *TLR-mDC*: R-848, IFN-γ, TNF-α and PGE_2_). Production of the T_h_1-polarizing cytokine IL-12p70 was assessed by ELISA after a 24-hr coculture of mDCs and CD40L-expressing 3T3 fibroblasts, as specified in "Methods". Results are shown from 3-9 independent experiments, each symbol expressing the mean of triplicate ELISA values obtained from one individual donor. The horizontal bars represent the mean IL-12p70 production in pg/mL per condition (*, *P *= 0.02).

As shown in table 2, neither IL-15 DCs nor IL-4 DCs were capable of primary IL-12p70 production. Since DC-mediated release of IL-12p70 upon CD40-CD40L signalling is considered to be more important than its primary production, we performed coculture experiments with DCs and CD40L-expressing 3T3 mouse fibroblast cells to mimic the *in vivo *CD40-CD40L molecular interaction between DCs and T-lymphocytes ("signal-3 assay"). As depicted in figure 5, no bioactive IL-12p70 could be detected in the coculture supernatants of conventionally matured IL-4 and IL-15 DCs (cc-mDC). By contrast, IL-15 DCs were capable of secreting detectable amounts of IL-12p70 upon TLR7/8 triggering (TLR-mDC). A more prominent, albeit heterogeneous, increment in IL-12p70 production was found after long-term culture of TLR7/8-matured IL-15 DCs (Figure 5). In addition, we observed that the primary culture supernatants of TLR7/8-matured IL-15 DCs contained high levels of IFN-γ, as opposed to conventionally matured IL-4 and IL-15 DCs. The IFN-γ secretion by TLR7/8-matured IL-15 DCs was independent of the duration of culture (Table 2; IFN-γ short-term *vs. *long-term TLR-mDC: *P *= 0.17).

The cytokine profile of IL-15 DCs was further explored by analyzing the release of T_h_2-related cytokines. As shown in table 2, no relevant amounts of IL-4, IL-5 and IL-10 could be detected in the culture supernatants of IL-15 DCs and IL-4 DCs (Table 2).

Moreover, signalling through TLR7/8 (TLR-mDC) resulted in the induction of high levels of TNF-α and IL-6 (Table 2). The production of the latter cytokine was substantially more pronounced in TLR-mDC as compared to cc-mDC. No statistically significant differences were found between short-term and long-term cultured TLR7/8-activated IL-15 DCs regarding their capacity to produce pro-inflammatory cytokines.

### Efficient induction of viral antigen-specific CD8^+ ^T cell responses by IL-15 DCs

**Figure 6:**
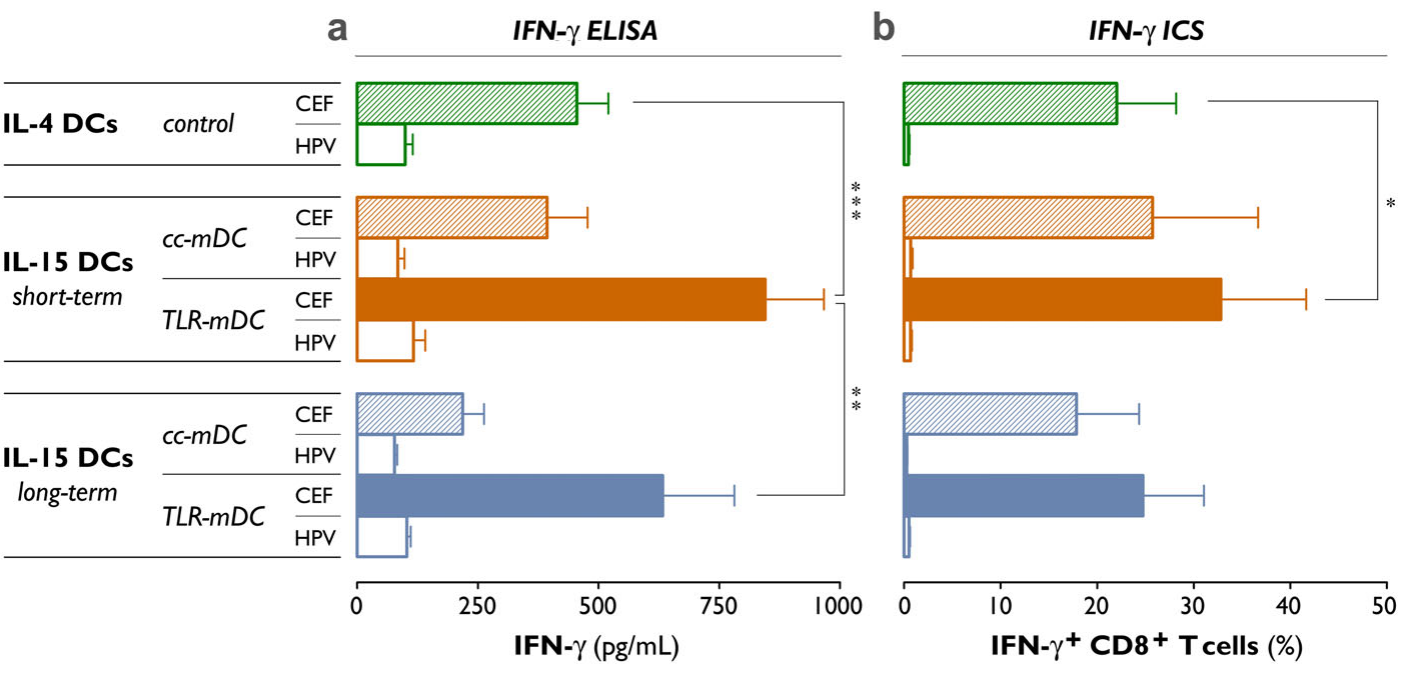
**Induction of viral antigen-specific CD8^+ ^T cell responses**. As described previously, short-term and long-term cultured IL-15 DCs were matured using two different maturation cocktails (*cc-mDC*: TNF-α, IL1β, IL-6 and PGE_2_; *TLR-mDC*: R-848, IFN-γ, TNF-α and PGE_2_). Conventionally matured IL-4 DCs were used as a control (control IL-4 DCs). The mDCs were harvested, pulsed with a pool of cytomegalovirus-, Epstein-Barr virus- and influenza a virus (CEF)-derived peptides, and cocultured with autologous PBLs for 7 days. Viral antigen-specific CD8^+ ^T cell responses were determined after this 7-day period and a short restimulation with the CEF peptide pool (CEF; filled bars). As specified in "Methods", the antigen-specific production of IFN-γ was assessed using two techniques: **(a) **ELISA to detect the amount of IFN-γ produced after restimulation (pg/mL) and **(b) **ICS to determine the % of IFN-γ^+ ^CD8^+ ^T cells. The non-specific IFN-γ release in response to restimulation with an irrelevant HPV peptide pool is shown (HPV; unfilled bars). Results are expressed as mean ± SEM of three independent experiments (*, *P *= 0.03; **, *P *= 0.006; ***, *P *< 0.001).

In order to determine their capacity to present viral antigens and to elicit antigen-specific CD8^+ ^T cell responses, mature DCs were pulsed with a peptide pool covering a panel of 32 MHC-I restricted T cell epitopes derived from the human cytomegalovirus, Epstein-Barr virus and influenza A virus (CEF), after which they were cocultured with autologous PBLs for 7 days. For all DC subsets tested, enhanced antigen-specific CD8^+ ^T cell responses were observed after antigen rechallenge with the CEF peptide pool as compared to an irrelevant peptide pool containing HPV_16 _E7 peptide sequences, which was included to evaluate the non-specific IFN-γ production (Figure 6).

We first examined whether the type of DC maturation cocktail (cc-mDC *vs. *TLR-mDC) had an impact on the capacity of IL-15 DCs to stimulate viral antigen-specific T cells. As shown in figure 6, a potent induction of antigen-specific CD8^+ ^T cell responses was observed when IL-15 DCs were exposed to the TLR7/8 ligand-based maturation cocktail (TLR-mDC). By contrast, restimulation of PBLs after prior coculture with conventionally matured IL-15 DCs (cc-mDC) resulted in much lower levels of secreted IFN-γ, as determined by ELISA (Figure 6a; short-term TLR-mDC *vs. *cc-mDC, *P *< 0.001; long-term TLR-mDC *vs. *cc-mDC, *P *= 0.004). This trend was also reflected by our ICS experiments, although the difference in number of IFN-γ^+ ^CD8^+ ^T cells between both maturation cocktails did not reach statistiscal significance (Figure 6b; short-term TLR-mDC *vs. *cc-mDC, *P *= 0.07; long-term TLR-mDC *vs. *cc-mDC, *P *= 0.06).

We next determined the effect of culture duration on the induction of antigen-specific CD8^+ ^T cell responses by IL-15 DCs. Short-term IL-15 DCs showed a distinctive superiority over their long-term cultured counterparts, regardless of the maturation cocktail used. This was evidenced by the increased ability of PBLs, stimulated by short-term cultured CEF-pulsed IL-15 DCs, to secrete IFN-γ upon antigen rechallenge. As shown in figure 6a, the antigen-specific IFN-γ release after CEF-restimulation of PBLs, was markedly increased in the short-term IL-15 DC subset. This phenomenon was found to be irrespective of the maturation protocol used (Figure 6a; short-term *vs. *long-term cc-mDC, *P *= 0.01; short-term *vs. *long-term TLR-mDC, *P *= 0.006). A parallel trend was observed in the number of IFN-γ^+ ^CD8^+ ^T cells (Figure 6b).

In general, a potent ability to induce recall immune responses could be attributed to short-term TLR7/8-matured IL-15 DCs. The superior T cell stimulation capacity of this DC subset was next verified against standard mature IL-4 DCs, and confirmed by the enhanced antigen-specific IFN-γ release and the higher frequencies of IFN-γ^+ ^CD8^+ ^T cells (Figure 6).

### mRNA electroporation is an effective strategy for antigen loading of IL-15 DCs

**Figure 7:**
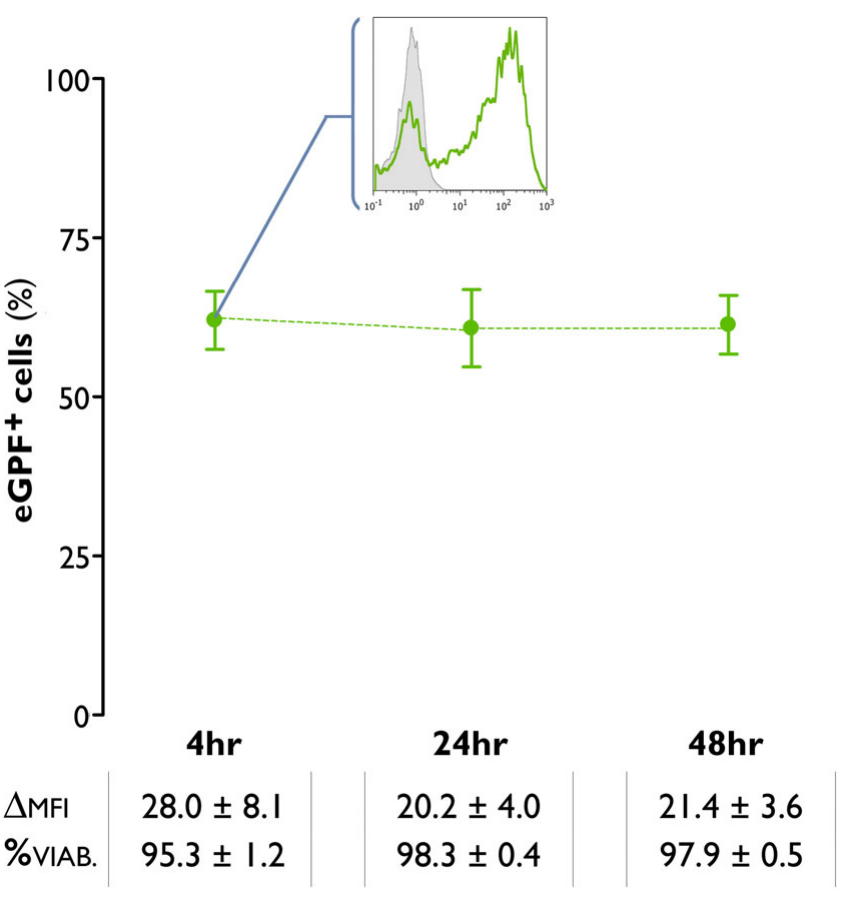
**mRNA transfectability of mature IL-15 DCs**. Monocytes were cultured for 2 days with GM-CSF + IL-15, followed by a 24-hr incubation with a TLR7/8 agonist-based maturation cocktail (*TLR-mDC*). The resultant mDCs were harvested and electroporated with mRNA encoding the enhanced green fluorescent protein (eGFP). The green dots represent the mean ± SEM percentages of eGFP^+ ^cells, as assessed by flow cytometry at different time points post-electroporation (4 hr, 24 hr, 48 hr). The insert shows a representative histogram overlay in which the flow cytometric eGFP expression 4 hr post-electroporation (green line histogram) is compared with the expression in a mock-electroporated negative control (grey-filled histogram). The values below indicate the delta MFI ± SEM of the eGFP expression (ΔMFI) and the mean ± SEM percentage of viable cells (%) at 4 hr, 24 hr and 48 hr following mRNA electrotransfection of IL-15 DCs (*n *= 5).

In view of the capacity of short-term TLR7/8-matured IL-15 DCs to mount cogent antigen-specific T cell responses after passive pulsing of antigen peptides, we next assessed whether mRNA electroporation could serve as an alternative antigen loading strategy of this DC subset. As a 'proof-of-principle' experiment for their mRNA transfectability, short-term TLR7/8-matured IL-15 DCs were electroporated in the presence or absence of eGFP mRNA. The mRNA electroporation efficiency was assessed by flow cytometry, showing stable transgene eGFP expression at different time points post-electroporation (4 hr, 24 hr, 48 hr). Percentages of eGFP^+ ^cells and their respective fluorescence intensities are shown in Figure 7. Besides the high transfection efficiency, electrotransfection of IL-15 DCs had no major impact on cell viabilities measured 4 hr, 24 hr and 48 hr after mRNA electroporation. Cell viability data are presented in figure 7 (Figure 7).

**Figure 8:**
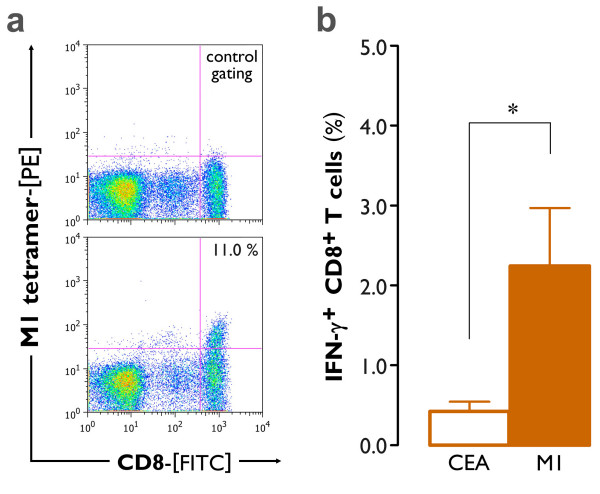
**Induction of antigen-specific CD8^+ ^T cell responses by mRNA-electroporated mature IL-15 DCs**. Short-term cultured IL-15 DCs were matured with our TLR7/8 agonist-based maturation cocktail (*TLR-mDC*), electroporated with mRNA encoding the influenza virus matrix protein M1 and cocultured with autologous PBLs for 6 days. **(a) **The expansion of M1-tetramer binding CD8^+ ^T cells was determined by flow cytometry. The lower dot plot represents the observed percentage of M1-tetramer^+ ^CD8^+ ^T cells in one representative donor (*n *= 4; mean ± SEM percentage of M1-tetramer^+ ^CD8^+ ^T cells: 4.4 ± 2.9). Correct positioning of the M1-tetramer^+ ^CD8^+ ^gate was defined by the respective negative control, as exemplified in the upper dot plot. **(b) **Simultaneously, a fraction of the PBL was harvested and stimulated with an irrelevant HLA-A*0201-restricted peptide (CEA) or rechallenged with the immunodominant influenza matrix protein (M1). The mean ± SEM percentage of antigen-specific IFN-γ^+ ^CD8^+ ^T cells was determined by ICS, as specified in the "Methods" section (*n *= 4; *, *P *= 0.03).

After the initial demonstration of their mRNA transfectability, we subsequently determined whether IL-15 DCs were able to elicit an antigen-specific cellular immune response after electroporation of antigen-encoding mRNA. For this purpose, PBLs from HLA-A*0201^+ ^healthy blood donors were exposed to autologous short-term cultured mature IL-15 DCs (TLR-mDC), that were electroporated as described above with mRNA encoding the influenza virus matrix protein M1. After one week of coculture, expansion of M1_(GILGFVFTL) _tetramer-positive CD8^+ ^T cells could be demonstrated in 3 out of 4 donors (Figure 8a). To confirm the findings of the tetramer staining, PBLs were restimulated with the HLA-A*0201-restricted peptides M1 (positive control) and CEA (negative control). After 4 hr of selective antigen rechallenge (M1), IFN-γ^+ ^CD8^+ ^T cells could be observed as shown in Figure 8b. Stimulation with the irrelevant CEA peptide confirmed the antigen specificity of the observed immune responses (Figure 8b; M1 *vs. *CEA, *P *= 0.03).

## Discussion

The current standard to generate DCs for use in clinical trials consists of a one-week, two-step culture protocol in which monocyte-derived DCs are first differentiated in the presence of GM-CSF and IL-4, and subsequently matured with a combination of pro-inflammatory cytokines [8,34]. In the present study, we established a novel protocol for the generation of monocyte-derived DCs by implementing the following modifications: *(1) *short-term culture for 2-3 days instead of 7 days, *(2) *alternative differentiation in the presence of GM-CSF and IL-15 (IL-15 DCs) and *(3) *alternative maturation induction through engagement of the TLR7/8 signalling pathway.

The modified protocol described here proved feasible for rapidly generating stimulatory and migratory DCs without any detrimental effects on cell viability and function. Neither differentiation with IL-15 nor TLR7/8 triggering had a negative influence on cell viability (data not shown). IL-15 DCs displayed a typical DC morphology already after 2-3 days of *in vitro *culture. As compared to standard IL-4 DCs, however, we observed that IL-15 DCs still retained some CD14 on their cell surface which, together with the lower expression levels of CD1a and CD209 (DC-SIGN), points to a less differentiated DC phenotype. This observation seemed unrelated to the duration of IL-15 DC culture, since long-term cultured IL-15 DCs expressed even higher levels of CD14 as compared to their short-term cultured counterparts. Previous studies have shown that replacement of IL-4 by IL-15 switches the differentiation of monocytes from 'genuine' monocyte-derived DCs to cells with a complex LC-like phenotype [22,23,40,41]. This finding has fuelled the interest in alternative differentiation of monocyte-derived DCs by IL-15, since LC-like DCs have been advocated as ideal cellular vaccine vehicles in view of their potent antigen-presenting capacity [4,42]. In the present study, we confirmed the Langerin (CD207)-positivity of IL-15 DCs [22,23] and showed that CD207 upregulation is already maximal after short-term culture.

Another intriguing phenotypic finding was that a fraction of IL-15 DCs expresses CD56, a marker with predominant expression on natural killer (NK) and NK-T cells [43]. In this regard, IL-15 DCs bear phenotypic similarity with monocyte-derived DCs generated in the presence of GM-CSF and IFN-γ (IFN-DCs). A subset of IFN-DCs was recently identified as being CD56-positive and endowed with endogenous cytotoxic activity, mediated by TNF-α-related apoptosis-inducing ligand (TRAIL) [43-45]. It is not completely speculative to draw a parallel between these IFN-DCs and IL-15 DCs, since there is evidence that type I interferons regulate IL-15 expression, suggesting a close relationship between both cytokines. In view of these data, it might be of particular interest to further elaborate on the phenotypic and potential functional resemblance of IFN-DCs and IL-15 DCs.

While it induces full phenotypic maturation in conventional IL-4 DCs, we observed that IL-15 DCs exhibit a suboptimal phenotype upon maturation induction with the widely adopted pro-inflammatory cytokine cocktail (cc-mDC). This was exemplified by the lower expression levels of the DC maturation marker CD83 and of vital costimulatory molecules such as CD80 and CD86. These phenotypic differences indicate that the results obtained with the classic maturation cocktail in IL-4 DCs cannot necessarily be extrapolated to IL-15 DCs.

Upon TLR activation, however, IL-15 DCs undergo an efficient maturation program and reach acceptable levels of CD83, CD70, CD80 and CD86; their phenotype appears close to that of fully mature IL-4 DCs, despite a distinct expression of CD83. The functional relevance of CD70 expression on the cell surface of TLR7/8-matured IL-15 DCs should be stressed, since CD70^+ ^DCs favour T_h_1 immunity via the CD70-CD27 signalling pathway in an IL-12p70-independent fashion [46].

We next examined several functional endpoints to which IL-15 DCs must conform in order to be a valid immunotherapeutic vaccine candidate. Migration of DCs to secondary lymphoid organs is generally considered a *conditio sine qua non *for the success of DC-based immunotherapy [47]. The migratory potential of IL-15 DCs has been sparsely investigated until present, with only one prior report demonstrating their migratory responsiveness to the CCR6 ligand CCL20 [22]. However, acquisition of CCR7 upon maturation is one of the critical factors involved in effective DC migration to the draining lymph nodes [6,48,49].

As expected, immature DCs showed absent expression of CCR7 and correspondingly failed to migrate in the direction of CCL21 in a standard Transwell™ migration assay, hence validating our experimental set-up [48]. While the classical combination of pro-inflammatory cytokines induces a migratory phenotype in standard IL-4 DCs (this study and [36,50]), IL-15 DCs are found to be refractory to this maturation cocktail. The low CCR7 expression and concomitant weak migratory potential of conventionally matured IL-15 DCs could be, at least in part, explained by their less mature phenotype, as reflected by the relative low expression of CD83. In contrast, TLR7/8 agonist-matured IL-15 DCs are capable of effective CCR7-mediated migration. This result is in line with recent studies, showing that the addition of PGE_2 _to the maturation protocol reinstates the migratory program affected by TLR signalling [32,33]. Our results clearly point to a superior migratory potential of short-term cultured TLR7/8-activated IL-15 DCs, which combine CCR7 expression with a migratory activity close to that of standard mature IL-4 DCs.

Besides possessing strong migratory properties, production of T_h_1-polarizing and pro-inflammatory cytokines is considered to be another characteristic of immunostimulatory DCs. Dendritic cell-mediated production of IL-12p70 upon T cell encounter in the lymph nodes is regarded as a decision step in the induction of a desired T_h_1 immune response [10]. Absent IL-12p70 release is a major barrier to effective immunotherapy, which could be circumvented by modifying the current *in vitro *DC maturation protocol [32,51]. As mentioned previously, the combination of TNF-α, IL-1β, IL-6 and PGE_2 _has been implemented as the standard maturation cocktail in most DC vaccine trials, despite its well-known drawback of hampering IL-12p70 production [38,39]. Analogous to conventional IL-4 DCs, we observed no overt IL-12p70 release by IL-15 DCs matured with the pro-inflammatory cytokine cocktail. Conversely, TLR7/8-activated IL-15 DCs are able to produce detectable amounts of IL-12p70 after mimicking *in vivo *T cell encounter with CD40L-expressing fibroblasts. It should be noted that typical high IL-12p70 levels could not be attained after activation of the TLR7/8 pathway in IL-15 DCs. This may be due to an intrinsic inability of IL-15 DCs to produce IL-12, as had been previously suggested [23]. The inclusion of PGE_2 _in our maturation cocktail provides another possible explanation. In contrast to recent studies [32,33], we found that exposure to PGE_2_clearly suppresses the IL-12p70 release by TLR7/8-matured IL-15 DCs (data not shown). However, the physiological relevance of this limited IL-12p70 production capacity is questionable for several reasons. First and foremost, it has been suggested that even minor amounts of IL-12p70 have a T_h_1-skewing influence on the immune response [52,53]. Thus one can hypothesize that the qualitative aspects (presence or absence of IL-12p70) are far more important than the quantitative. Within this context, it is also difficult to judge the significance of the more pronounced IL-12p70 release by long-term cultured TLR7/8-matured IL-15 DCs as opposed to their short-term counterparts. Secondly, IL-12p70 is an important but not exclusive signal for the induction of T_h_1 responses. Effective cellular immune responses can occur in the absence of functional IL-12p70, as has been exemplified in the case of Langerhans cells [42]. Thirdly, we were able to show that TLR7/8-activated IL-15 DC preparations contain high amounts of IFN-γ, which are likely derived from expanded NK cells in the IL-15 DC cultures [25]. A recent study by Hardy *et al. *has pointed out the pivotal role of contaminating IFN-γ producing NK cells in the induction of T_h_1 immunity by IL-15 DCs. As such, it might be speculated that NK cell-derived IFN-γ can partially replace IL-12p70 as a T_h_1-polarizing cytokine, thereby providing another mechanism by which IL-15 DCs can induce cellular immunity in an IL-12-independent fashion [25]. Fourthly, TLR7/8-matured IL-15 DCs express CD70, which contributes to IL-12-independent T_h_1 differentiation as described above [46]. Lastly, Dubsky *et al. *have recently elucidated that the enhanced potential of IL-15 DCs to induce cellular immune responses can be ascribed in part to membrane transpresentation of the T_h_1-polarizing cytokine IL-15 [24].

Efficient antigen presentation is another prerequisite that DCs must fulfill in order to be considered for implementation in DC-based immunotherapy protocols. Previous studies convincingly showed that IL-15 DCs are highly capable of inducing antigen-specific T cell responses in both viral and tumor antigen models [22,24,41]. It has been put forward that IL-15 DCs have an optimal antigen-presenting capacity; a recent study by Dubsky *et al. *has emphasized their potent ability to prime and expand high-avidity tumor antigen-specific CD8^+ ^cytotoxic T lymphocytes [24]. Our study extend these findings in several important respects. In the first place, activation of IL-15 DCs with a TLR7/8 stimulus appears to result in enhanced antigen-specific T cell responsiveness compared to maturation with a standard combination of pro-inflammatory cytokines. This observation should be interpreted together with the other effects of the two studied maturation cocktails on IL-15 DCs. On the basis of their phenotypic profile and their impaired ability for CCR7-driven migration and cytokine production, it can be hypothesized that IL-15 DCs are relatively inert to classical maturation stimuli, thereby providing an explanation for their inferior capacity to induce antigen-specific T cell responses. Since fully mature, immunostimulatory DCs are required for successful cancer immunotherapy, the clinical use of cytokine cocktail-matured IL-15 DCs cannot be recommended. Conversely, TLR7/8-matured IL-15 DCs showed a strong T cell stimulation capacity. This is particularly true for short-term cultured IL-15 DCs, which demonstrated a clear superiority over their long-term cultured counterparts and over conventional IL-4 DCs with regard to the induction of antigen-specific CD8^+ ^T cell responses.

Taken together, short-term cultured and TLR7/8-matured IL-15 DCs best meet the imposed requirements to be recognized as immunostimulatory DCs. Not only do they possess an accurate phenotype as described above, they also combine strong migratory properties with some degree of IL-12p70 production and, most importantly, with a potent ability to promote antigen-specific T cell responses. Despite a more pronounced secondary production of IL-12p70, their long-term cultured counterparts behaved inferior with respect to migration and T cell stimulation capacity. In addition, reducing the time of DC culture facilitates the still arduous and costly process of *ex vivo *DC generation. Since short-term culture and TLR7/8-induced maturation of IL-15 DCs was considered as the "best-fit" approach to generate immunostimulatory DCs, the last part of our study was dedicated to the mRNA electroporability of this DC subset. Electroporation of mRNA is being increasingly applied in clinical vaccination trials as an elegant strategy for antigen loading of DCs [54,55]. The attractiveness of this technique is based on the fact that it overcomes several drawbacks of other antigen delivery methods, such as the biosafety issues posed by viral gene delivery or the need for genome integration and the related risk of insertional mutagenesis associated with DNA transfection. In contrast to exogenous peptide pulsing, mRNA electroporation does not require prior knowledge of the HLA restriction characteristics of the antigen epitopes nor the need for HLA-matched donor DCs [55-57]. Here we demonstrate for the first time the mRNA transfectability of IL-15 DCs. Short-term cultured TLR7/8-matured IL-15 DCs show accurate transgene expression after eGFP mRNA electroporation, consistent with prior studies on TLR7/8-mediated DC maturation [31,58]. Moreover, the observation that M1 mRNA-electroporated IL-15 DCs are capable to induce influenza matrix protein M1-specific T cells further proves the feasibility and applicability of this method.

## Conclusions

In conclusion, we propose a novel approach for the generation of DCs, based on a combined strategy of *(1) *short-term culture of monocyte-derived DCs, *(2) *differentiation in the presence of IL-15 and *(3) *maturation using a TLR7/8 ligand-based cocktail. This integrative approach results in the generation of DCs that meet the phenotypic and functional endpoints for implementation in clinical vaccination trials.

## List of Abbreviations

cc-mDC: Conventional maturation cocktail (see Table 1 for details); CD40L: CD40 ligand; CEF: Cytomegalovirus, Epstein-Barr virus and influenza virus; DC(s): Dendritic cell(s); ΔMFI: Delta mean fluorescence intensity (MFI specific antibody - MFI isotype control); EDTA: Ethylenediaminetetraacetic acid; eGFP: Enhanced green fluorescent protein; FITC: Fluorescein isothiocyanate; GM-CSF: Granulocyte macrophage colony-stimulating factor; HLA: Human leukocyte antigen; HPV: Human papilloma virus; ICS: Intracellular staining; IFN: Interferon; IL: Interleukin; IMDM: Iscove's Modified Dulbecco's Medium; LC: Langerhans cell; mAb: Monoclonal antibody; MHC: Major histocompatibility complex; MIA: Multiplex immunoassay; NK: Natural killer; PBLs: Peripheral blood lymphocytes; PBMC(s): Peripheral blood mononuclear cell(s); PBS: Phosphate-buffered saline; PE: Phycoerythrin; PerCP: peridinin chlorophyll protein; PGE_2_: Prostaglandin E_2_; PI: Propidium iodide; RPMI: Roswell Park Memorial Institute; SD: standard deviation; SEM: standard error of the mean; T_h_1: T helper type 1; TLR: Toll-like receptor; TLR-mDC: TLR7/8 agonist-based maturation cocktail (see Table 1 for details); TNF: Tumor necrosis factor.

## Competing interests

The authors declare that they have no competing interests.

## Authors' contributions

SA designed the study, performed the statistical analysis and drafted the manuscript. ELJMS contributed to the study design and has been involved in drafting the manuscript. NC participated in the experimental work. HG, ZNB and VFIVT participated in the design of the study and critically revised the manuscript for important intellectual content. All authors have read and approved the final version of the manuscript.

## Supplementary Material

Additional file 1 **Phenotype of mature IL-15 DCs**. Cell surface expression of CD40, CD70, CD80, CD83, CD86 and CD209 on mature DCs (short-term cultured IL-15 DCs, long-term cultured IL-15 DCs and IL-4 DCs). Dendritic cell maturation was induced using a pro-inflammatory maturation cocktail (cc-mDC; see Table 1 for details) or a TLR7/8 ligand-containing mixture (TLR-mDC; see Table 1 for details). Flow cytometry results of 4 independent experiments are expressed as mean ± SEM percentage of positive cells (% pos.) and as delta MFI ± SEM (ΔMFI), according to the protocol described in "Methods". Click here for file
