# Supplementary material for: Short-term cultured, interleukin-15 differentiated dendritic cells have potent immunostimulatory properties
Source: J Transl Med. 2009 Dec 18;7:109. doi: 10.1186/1479-5876-7-109 (PMC2807857; doi:10.1186/1479-5876-7-109)
Supplement: Additional file 1 — Phenotype of mature IL-15 DCs. Cell surface expression of CD40, CD70, CD80, CD83, CD86 and CD209 on mature DCs (short-term cultured IL-15 DCs, long-term cultured IL-15 DCs and IL-4 DCs). Dendritic cell maturation was induced using a pro-inflammatory maturation cocktail (cc-mDC; see Table 1 for details) or a TLR7/8 ligand-containing mixture (TLR-mDC; see Table 1 for details). Flow cytometry results of 4 independent experiments are expressed as mean ± SEM percentage of positive cells (% pos.) and as delta MFI ± SEM (ΔMFI), according to the protocol described in "Methods". [file 1479-5876-7-109-S1.DOC]

| **Additional file 1.** Phenotype of mature IL-15 DCs. | | | | | | | | |
| --- | --- | --- | --- | --- | --- | --- | --- | --- |
|  | | | | | | | | |
|  |  | **short-term IL-15 DCs** | |  | **long-term IL-15 DCs** | |  | **IL-4 DCs** |
|  |  | ***cc-mDC*** | ***TLR-mDC*** |  | ***cc-mDC*** | ***TLR-mDC*** |  | ***cc-mDC*** |
|  | | | | | | | | |
| ***CD40*** | *% pos.* | 43.8 ± 2.0 | 70.8 ± 3.5 |  | 62.7 ± 11.4 | 75.1 ± 4.9 |  | 79.2 ± 2.4 |
|  | *Δ_MFI_* | 4.1 ± 0.6 | 8.9 ± 1.5 |  | 8.6 ± 4.1 | 8.0 ± 1.3 |  | 7.5 ± 0.5 |
|  | | | | | | | | |
| ***CD70*** | *% pos.* | 36.2 ± 6.6 | 63.6 ± 2.5 |  | 17.0 ± 8.2 | 63.9 ± 6.2 |  | 96.2 ± 0.8 |
|  | *Δ_MFI_* | 4.2 ± 1.2 | 10.6 ± 1.6 |  | 0.7 ± 0.8 | 8.3 ± 2.0 |  | 32.7 ± 3.3 |
|  | | | | | | | | |
| ***CD80*** | *% pos.* | 79.2 ± 5.0 | 95.8 ± 0.8 |  | 90.4 ± 1.7 | 98.8 ± 0.3 |  | 98.1 ± 0.5 |
|  | *Δ_MFI_* | 27.3 ± 5.1 | 109.4 ± 13.1 |  | 25.8 ± 3.2 | 115.4 ± 11.3 |  | 172.0 ± 16.5 |
|  | | | | | | | | |
| ***CD83*** | *% pos.* | 44.3 ± 5.8 | 72.7 ± 4.2 |  | 53.6 ± 2.5 | 80.7 ± 4.7 |  | 97.8 ± 0.5 |
|  | *Δ_MFI_* | 5.5 ± 1.2 | 13.1 ± 2.5 |  | 3.3 ± 0.5 | 16.1 ± 6.2 |  | 135.4 ± 8.0 |
|  | | | | | | | | |
| ***CD86*** | *% pos.* | 96.8 ± 0.9 | 98.9 ± 0.3 |  | 87.1 ± 3.6 | 98.3 ± 0.8 |  | 99.9 ± 0.1 |
|  | *Δ_MFI_* | 163.6 ± 30.9 | 351.6 ± 21.7 |  | 38.1 ± 13.0 | 141.8 ± 16.6 |  | 308.8 ± 17.3 |
|  | | | | | | | | |
| ***CD209*** | *% pos.* | 62.8 ± 3.6 | 66.0 ± 4.3 |  | 63.2 ± 3.9 | 50.4 ± 5.7 |  | 97.0 ± 0.3 |
|  | *Δ_MFI_* | 14.2 ± 2.3 | 18.7 ± 3.2 |  | 13.8 ± 3.0 | 8.2 ± 1.8 |  | 70.6 ± 3.8 |
|  | | | | | | | | |
